# Telemedicine With Wearable Technologies in Patients Undergoing Hematopoietic Cell Transplantation and Chimeric Antigen Receptor T-Cell Therapy (TEL-HEMATO Study): Prospective Noninterventional Single-Center Study

**DOI:** 10.2196/55918

**Published:** 2024-06-04

**Authors:** Lidia Hurtado, Melinda Gonzalez Concepcion, Aida Flix-Valle, Marina Ruiz-Romeo, Sonia Gonzalez-Rodriguez, Marta Peña, Annalisa Paviglianiti, Maria Angeles Pera Jambrina, Anna Sureda, Cristian Ochoa-Arnedo, Alberto Mussetti

**Affiliations:** 1 Clinical Hematology Department Institut Català d'Oncologia Hospital Duran i Reynals Barcelona Spain; 2 Department of Fundamental and Clinical Nursing Faculty of Nursing Bellvitge Campus, Universitat de Barcelona Barcelona Spain; 3 Psychooncology and Digital Health Research Group Barcelona Spain; 4 ICOnnecta't Digital Health Program and Psycho-Oncology Service Institut Català d'Oncologia Barcelona Spain; 5 Department of Clinical Psychology and Psychobiology Universitat de Barcelona Barcelona Spain; 6 Institut d'Investigació Biomèdica de Bellvitge Barcelona Spain; 7 Department of Medicine Unit of Endocrinology and Diabetes Università Campus Bio-Medico di Roma Rome Italy

**Keywords:** hematology, hematopoietic cell transplantation, telemedicine, wearables, chimeric antigen receptor T, CART, wearable, hematopoietic, transplantation, transplant, pilot study, hematological, HCT, telehealth, therapy, device, quality of life, digital health, smartphone, app, patient, teenager, youth, noninterventional

## Abstract

**Background:**

Patients with hematological malignancies receiving hematopoietic cell transplantation (HCT) or chimeric antigen receptor (CAR) T-cell therapy are at risk of developing serious clinical complications after discharge.

**Objective:**

The aim of the TEL-HEMATO study was to improve our telehealth platform for the follow-up of patients undergoing HCT or CAR T-cell therapy during the first 3 months after discharge with the addition of wearable devices.

**Methods:**

Eleven patients who received autologous (n=2) or allogeneic (n=5) HCT or CAR T-cell therapy (n=4) for hematological malignancies were screened from November 2022 to July 2023. Two patients discontinued the study after enrollment. The telehealth platform consisted of the daily collection of vital signs, physical symptoms, and quality of life assessment up to 3 months after hospital discharge. Each patient received a clinically validated smartwatch (ScanWatch) and a digital thermometer, and a dedicated smartphone app was used to collect these data. Daily revision of the data was performed through a web-based platform by a hematologist or a nurse specialized in HCT and CAR T-cell therapy.

**Results:**

Vital signs measured through ScanWatch were successfully collected with medium/high adherence: heart rate was recorded in 8/9 (89%) patients, oxygen saturation and daily steps were recorded in 9/9 (100%) patients, and sleeping hours were recorded in 7/9 (78%) patients. However, temperature recorded manually by the patients was associated with lower compliance, which was recorded in 5/9 (55%) patients. Overall, 5/9 (55%) patients reported clinical symptoms in the app. Quality of life assessment was completed by 8/9 (89%) patients at study enrollment, which decreased to 3/9 (33%) at the end of the third month. Usability was considered acceptable through ratings provided on the System Usability Scale. However, technological issues were reported by the patients.

**Conclusions:**

While the addition of wearable devices to a telehealth clinical platform could have potentially synergic benefits for HCT and CAR T-cell therapy patient monitoring, noncomplete automation of the platform and the absence of a dedicated telemedicine team still represent major limitations to be overcome. This is especially true in our real-life setting where the target population generally comprises patients of older age with a low digital education level.

## Introduction

In the ever-evolving landscape of health care, technological advancements are ushering in a new era of patient-centric care. For individuals who have undergone hematopoietic cell transplantation (HCT) or chimeric antigen receptor (CAR) T-cell therapy, the posttreatment journey often involves continuous monitoring and timely intervention. Patients are susceptible to a myriad of complications (eg, infections, cytokine release syndrome, neurotoxicity, graft-versus-host disease), necessitating vigilant monitoring. Traditional posttransplant care models involve frequent hospital visits, which can be both logistically challenging and emotionally taxing for patients.

Telemedicine platforms enable the secure and real-time communication between patients and health care providers, allowing for remote consultations, symptom management, and medication adherence monitoring [1]. This ensures that medical professionals can intervene promptly when needed. The incorporation of wearable devices further enhances this approach, facilitating the detection of subtle changes in health parameters and enabling timely intervention [2]. This paradigm shift in health care delivery not only improves the overall patient experience but could also potentially contribute to better outcomes [3]. Patients can regain a sense of control over their health, fostering a proactive approach to recovery. Moreover, the integration of telemedicine and wearables aligns with the broader trend of precision medicine, allowing for tailored interventions based on real-time patient data.

However, there is still no standardized or preferred platform for telemedicine in the hematological setting. The results of existing studies are heterogeneous and in most cases the telemedicine platform consisted of only phone or video calls with patients. The majority of existing telehealth systems are not validated as safe for the patient or capable of detecting the patient’s physical status, which is especially relevant in the setting of patients at high risk of medical acute complications.

In this study, we present the results of assessment of the use of an improved telehealth platform with the addition of wearable devices for the early clinical monitoring of patients during follow-up of HCT and CAR T-cell therapy.

## Methods

### Participants

All patients were screened and enrolled at the Hematology Department of the Catalan Institute of Oncology, Hospital Duran i Reynals (Hospitalet de Llobregat, Barcelona, Spain), between November 2022 and July 2023. Inclusion criteria were (1) aged>18 years, (2) received autologous or allogeneic HCT or CAR T-cell therapy for hematological malignancies, (3) owning a smartphone compatible with the study app ICOnnecta’t (Android or iOS systems), (4) and signed the informed consent form. Exclusion criteria were (1) insufficient social or family support and (2) uncontrolled disease at the time of HCT or CAR T-cell therapy.

### Ethical Considerations

The study was approved by the Ethics Committee of the Bellvitge University Hospital (reference PR078/22) and was conducted in accordance with the Spanish Medical Research with Human Subjects law. Informed consent was obtained from the study participants. Patients could discontinue participation at any time without penalty. Data are stored on secure servers and only those involved in this study have access to the data. All methods were performed in accordance with relevant guidelines and regulations.

### Study Design and Platform

**Figure 1:**
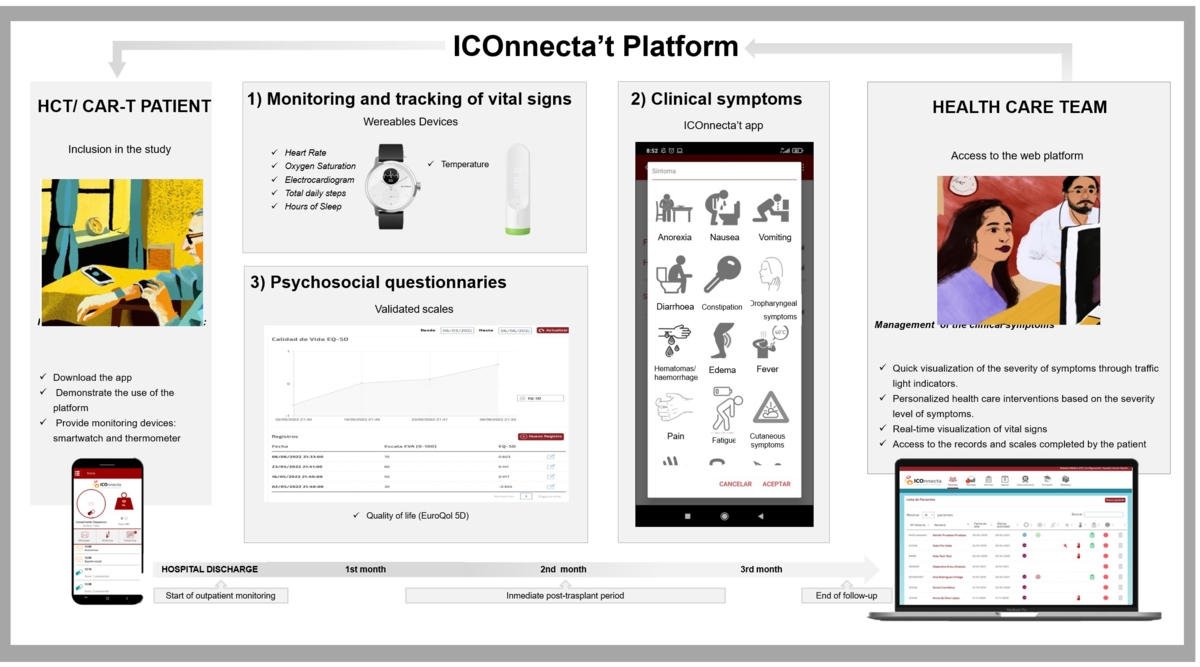
Schematic representation of the TEL-HEMATO study platform. CAR-T: chimeric antigen receptor T-cell therapy; HCT: hematopoietic cell transplantation.

This was a prospective, noninterventional, single-center study (TEL-HEMATO study). Enrollment was performed 1-3 days before hospital discharge. At this time, patients were provided with a clinically validated smartwatch (ScanWatch, Withings) and a digital thermometer (Thermo, Withings). The study app was installed on the patient’s smartphone. Data collected through wearable devices and the study app were transmitted to an online platform for daily review by health care workers (nurses or hematologists) (Figure 1).

Wearable devices allowed for the collection of the following vital signs: heart rate (every 10 minutes), peripheral blood oxygen saturation (every 10 minutes at night), body temperature (every 24 hours, collected actively by the patient), total daily steps (every 24 hours), and sleeping hours (every 24 hours). The ICOnnecta’t app was used for the collection of sociodemographic data (at study initiation), clinical symptoms, and psychological variables [4]. Clinical symptoms (anorexia, nausea, vomiting, diarrhea, constipation, oropharyngeal symptoms, edema, fever, cutaneous symptoms, fatigue, presence of hematomas or hemorrhage, and pain) were collected through the smartphone app whenever the patient experienced a symptom and recorded it on the app throughout the study period. Quality of life (QoL) was assessed using the EQ-5D-3L questionnaire every 28 days [5]. The usability of the platform was assessed through the System Usability Scale (SUS) [6], which comprises 10 items rated on a Likert-type scale, ranging from 1 (strongly disagree) to 5 (strongly agree).

Programmed alarms were set in case any of the following vital signs or symptoms were detected: fever>38 °C, oxygen saturation<95%, heart rate <50 or >100 beats per minute (bpm), and/or severe symptoms as defined by standard clinical criteria (see Table S1 in Multimedia Appendix 1). In case of alarm activation, the nurse or hematologist would assess the necessity for an in-person visit and determine the most suitable clinical management strategy. The physiotherapist of the study was responsible for reviewing the daily steps reached for the patients and performed a monthly call with the aim of motivating patients to reach 7500 steps/day.

Outpatient monitoring started from the day of hospital discharge (±2 days) and continued up to 3 months or until considered clinically needed by the treating physician or nurse, whichever came first. At the end of the recruitment period, patients were contacted by phone and were asked to fill out the usability scale assessment.

### Primary and Secondary Endpoints

The primary endpoint of the study was the real-life feasibility of the use of the telehealth platform. Secondary endpoints were: (1) vital signs collected through the use of wearable devices, (2) symptoms collected during the study period, and (3) psychosocial variables collected through the study app.

## Results

### Patient Characteristics

A total of 11 participants were enrolled in the pilot study. Two patients discontinued the study after enrollment (one due to technical issues with the smartphone and the other due to a change of treating center). No patients died during the study period. The median age of the participants was 54 (range 20-64) years and 54% (6/11) were female. Allogeneic HCT was the most common therapy (5/11, 45%; a total of 31 allogeneic HCTs were performed during the study period), followed by CAR T-cell therapy (4/11, 36%; a total of 9 patients underwent CAR T-cell therapy during the study period) and autologous HCT (2/11, 18%; a total of 36 autologous HCTs were performed during the study period). Among the 11 patients included in the study, 3 (27%) exhibited medium psychosocial complexity and 1 (9%) displayed high psychosocial complexity, as defined by Gil et al [7]. The main sociodemographic and clinical characteristics of the 11 patients enrolled in the study are shown in Table 1. Platform results are reported excluding the 2 patients who withdrew their participation from the study.

**Table 1:** Demographic and clinical characteristics of the patients included in the study (N=11).

Characteristics			Value
Age (years), median (range)			54 (20-64)
Female sex, n (%)			6 (54)
**Type of malignancy, n (%)**			
	Acute myeloid leukemia		1 (9)
	Acute lymphoblastic leukemia		2 (18)
	**Non-Hodgkin lymphoma**		
		Diffuse large B-cell lymphoma	4 (36)
		T-cell lymphoma	1 (9)
		Myelodysplastic syndrome	1 (9)
		Multiple myeloma	2 (18)
**Therapy, n (%)**			
	Autologous HCT^a^		2 (18)
	Allogeneic HCT		5 (45)
	CAR-T^b^ therapy		4 (36)
Time between day 0 and inclusion in the ICOnnecta’t platform (days), median (range)			11 (0-18)
Duration of monitoring on the platform (days), median (range)			125 (6-279)
**Social-family indicators, n (%)^c^**			
	Caregiver issues		0 (0)
	Family dynamic issues		0 (0)
	Practical or economic issues		0 (0)
	Has vulnerable dependents		2 (18)
	Immigration-related challenges		0 (0)
	Challenges related to the situation of a displaced patient/out of area		2 (18)
	Presented to Psychosocial Committee		0 (0)
**Psychosocial complexity level, n (%)**			
	Low		7 (64)
	Medium		3 (27)
	High		1 (9)

^a^HCT: hematopoietic cell transplantation.

^b^CAR-T: chimeric antigen receptor T cell.

^c^Based on data of 9 patients, excluding the 2 patients who withdrew their participation.

### Vital Signs Monitoring

Temperature was recorded in 5/9 (55%) patients during the first month, 4/9 (44%) patients during the second month, and 4/9 (44%) patients during the third month. Temperature alarms were recorded 12 times only in one patient. Heart rate abnormalities were recorded 18,764 times (1144 times for <50 bpm and 17,620 for >100 bpm) in 8/9 (89%) patients throughout the study period. Oxygen saturation abnormalities were recorded 5884 times in 10/10 (100%) patients during the study period. The median daily steps per day was 1774 (range 1-5099) during the first month (100% of patients recorded), 3200 (range 1342-6660) during the second month (5/9, 55% of patients recorded), and 2749 (range 0-9707) during the third month (5/9, 55% of patients recorded). Only one of the 11 patients included in the study achieved the 7500 steps/day goal during the study period. The patients slept a median of 7.1 hours/day (range 5.4-8.1) during the first month (78% of patients recorded), 7.05 hours/day (range 4.3-8.7) during the second month (67% of patients recorded), and 7.1 hours/day (range 5.5-10.6) during the third month (55% of patients recorded).

### Symptoms Reporting

Overall, 5/9 (55%) patients reported a total of 26 symptoms during the study (Table 2). Among these symptoms, one was classified as severe (grade 3), one as moderate (grade 2), and 21 as mild (grade 1), with the remaining 3 symptoms reported as of no consequence (grade 0). The most commonly reported symptoms were vomiting, followed by fatigue and pain. Participants also reported experiencing nausea, constipation, and anorexia (Table 2).

**Table 2:** Summary of self-reported symptoms.

Symptom	Times reported	Severity			
		Grade 0	Grade 1	Grade 2	Grade 3
Vomiting	11	3	7	0	1
Fatigue	6	0	5	1	0
Pain	4	0	4	0	0
Nausea	2	0	2	0	0
Constipation	2	0	2	0	0
Anorexia	1	0	1	0	0

### Quality of Life

The mean QoL score was 0.633 (range 0-1) at study enrollment (recorded by 8/9 [89%] of patients), 0.800 (range 0.099-1.000) during the first month (recorded by 6/9 [67%] of patients), 0.833 (range 0.514-1.000) during the second month (recorded by 4/9 [44%] of patients), and 0.824 (range 0.648-1.000) during the third month (recorded by 3/9 [33%] of patients).

### Usability

The median length of monitoring on the platform was 125 (range 6-279) days. Overall, 7/9 (78%) patients completed the SUS (see Table S2 in Multimedia Appendix 1) at the end of the study period. Participants found the app relatively easy to use, with high scores given in areas such as feeling secure while using the app and the integration of different app functions. However, some participants expressed concerns about the complexity of the app and the need for technical support (Multimedia Appendix 2).

### Patient Satisfaction: Qualitative Data

User feedback on the app and wearable devices was predominantly positive. Features such as temperature monitoring and step count were widely appreciated for their utility and motivational impact. Although some users expressed initial challenges, prompt resolution contributed to a positive overall experience. Recommendations for enhancements included increased technical support, especially for those less technologically proficient. Despite varied usage of additional features, educational resources, and apps, there was unanimous satisfaction among users, reflecting their willingness to recommend the technology to others. The feedback emphasized the potential positive influence of these technologies in fostering behavioral changes and improving overall well-being. Detailed results can be found in Multimedia Appendix 2.

## Discussion

We tested the addition of wearable devices to improve the performance of a telehealth system for monitoring patients with hematological malignancies after treatment. Results from our previous study [8] showed that the use of self-reported outcomes in the setting of telemonitoring led to low compliance, with only 12 out of 21 patients capable of using the telehealth platform. Moreover, of the 12 patients enrolled in telemonitoring, the adherence was not ideal, especially for the collection of vital signs. In most cases, this was explained by technological issues and a low level of digital education. Moreover, self-reported monitoring of vital data could lead to inaccuracy and mistakes if performed by individuals other than health care personnel. The use of wearable devices could effectively automatize the reporting of health data and improve study compliance. Wearable use in these contexts has been under investigation in a few clinical studies globally, including for patients receiving CAR T-cell therapy in the outpatient setting [9].

With respect to the reporting of vital signs, we confirmed that the use of a clinically certified smartwatch can effectively collect a large amount of clinical data without the patient needing to play an active role. This was applicable for all of the variables collected automatically by the smartwatch (heart rate, oxygen saturation, daily steps, and sleeping hours). For these variables, data recording was generally higher than 70%. This result is in line with other clinical studies for patients with other conditions, including in the field of cardiology where the use of wearable devices has already proven to be effective and able to collect continuous data [3]. In contrast, temperature collection adherence was low, with data recorded for only 55% of patients during the first month and this rate was reduced to 44% at the end of the study period. This likely reflects the fact that temperature detection relies on the use of a second device and thus necessitates active intervention from the patient. Despite the patients stating that use of a thermometer was considered useful in the domiciliary setting, recording of temperature data on the platform was insufficient. Recently, preliminary results regarding the use of wearable devices for the monitoring of patients undergoing CAR T-cell therapy indicated higher adherence (>70%) for all vital signs, including temperature [10,11]. However, in both of these studies, temperature was recorded automatically and did not require any intervention from the patients.

Although the reporting of symptoms provided useful clinical information in our study, this was only performed by five patients. This could also reflect a lack of digital education or confidence in using a digital device to seek medical attention in older patients. The platform was able to collect the responses to questionnaires about QoL. However, the adherence in collecting these forms decreased over time from 89% at study enrollment to 33% after the third month. This drop in adherence suggests that patients are willing to use digital platforms until they perceive their clinical status to have improved. This was also confirmed by the personal comments of study participants. Similar results have been reported in another study, where only 37 out of 123 patients reported QoL data at the end of the study period [9].

There are several limitations of this study to consider when interpreting the results. First, the technological barrier was evident in this setting. The majority of patients, especially older patients, did not have sufficient digital experience to use the smartphone app in the appropriate manner. This led to inefficient data collection from the wearable devices or self-reported symptoms. For this reason, future studies should aim to achieve the complete automation of wearable devices for data transmission and a user-friendly interface. Second, the use of wearable devices generated a large amount of data for each patient. The interpretation of such data and assigning clinical significance to these data can be challenging and should be clearly discussed and set together with health care operators whenever creating a platform for the digital monitoring of patients [12]. Big data can generate confusion if the method of interpretation is not well defined and oriented to specific aims. Third, use of a telehealth platform generated a significant workload for the health care professionals involved in the study. The time allocated for clinical research exceeded what was planned before study initiation. It was not always possible to perform daily quality control of all the data collected from the platform due to insufficient time dedicated to investigation. In addition, it was not possible to perform the clinical monitoring during nights or holidays due to the absence of research personnel. Institutions and clinical research groups with interest in telehealth platforms should consider having sufficient and dedicated human resources for the implementation of a telemedicine team. This team should be available 24 hours a day 7 days a week. Finally, the limited number of patients included prevented us from performing a formal analysis of vital signs data in relation to HCT and CAR T-cell therapies. However, the aim of the study was to test the feasibility and real-life use of this improved telehealth platform. Therefore, the statistical analysis and clinical utility evaluation of the platform were beyond the scope of the study.

In conclusion, the addition of wearable devices to a telehealth platform for patients following HCT and CAR T-cell therapy has the potential to improve clinical monitoring. However, the lack of complete automation in patient monitoring, issues in interpreting the large amount of data generated, technological barriers, and the absence of a specific team dedicated to telehealth platforms still represent major issues that can preclude the widespread use of such technologies in daily clinical practice.

## Appendix

Multimedia Appendix 1 Symptom grading scale (Table S1) and usability acceptance scores (Table S2).

Multimedia Appendix 2 Subjective evaluations of user experience with the ICOnnecta’t system.

## Abbreviations

bpm: beats per minute
CAR: chimeric antigen receptor
HCT: hematopoietic cell transplantation
QoL: quality of life
SUS: System Usability Scale
