# Supplementary material for: Telemedicine With Wearable Technologies in Patients Undergoing Hematopoietic Cell Transplantation and Chimeric Antigen Receptor T-Cell Therapy (TEL-HEMATO Study): Prospective Noninterventional Single-Center Study
Source: JMIR Form Res. 2024 Jun 4;8:e55918. doi: 10.2196/55918 (PMC11185900; doi:10.2196/55918)
Supplement: Multimedia Appendix 1 [file formative_v8i1e55918_app1.docx]

**Multimedia Appendix 1**

**Table S1.** Symptom grading scale

|  | **Description** | **Intervention** |
| --- | --- | --- |
| **Grade 0** | No symptoms | None |
| **Grade 1** | Mild symptoms, not interfering with daily activities | Health education for symptom management |
| **Grade 2** | Moderate symptoms, causing some interference with daily activities | Health education for symptom management + nurse will contact within 24/48 hours. |
| **Grade 3** | Severe symptoms, significantly impacting daily activities | Instructions are provided for immediately contacting the nurse and/or for emergencies. |

**Table S2**. Usability Assessment Scores for the Application

| **Usability scale** | **Mean** |
| --- | --- |
| 1. I think I would like to use this application frequently. | 4.28/5.00 |
| 2. I think the application is unnecessarily complex. | 2.00/5.00 |
| 3. I think the application is easy to use. | 4.14/5.00 |
| 4. I think I would need technical support to be able to use the application. | 2.14/5.00 |
| 5. I think the different functions of the application are well integrated. | 4.42/5.00 |
| 6. I believe there is too much inconsistency in this application. | 1.57/5.00 |
| 7. I believe most people would learn to use this application quickly. | 4.00/5.00 |
| 8. I think the application is very difficult to use. | 1.85/5.00 |
| 9. I feel very confident using the application. | 4.71/5.00 |
| 10. I would need to learn many things before I could start using the application. | 2.71/5.00 |
