# Supplementary material for: Telemedicine With Wearable Technologies in Patients Undergoing Hematopoietic Cell Transplantation and Chimeric Antigen Receptor T-Cell Therapy (TEL-HEMATO Study): Prospective Noninterventional Single-Center Study
Source: JMIR Form Res. 2024 Jun 4;8:e55918. doi: 10.2196/55918 (PMC11185900; doi:10.2196/55918)
Supplement: Multimedia Appendix 2 [file formative_v8i1e55918_app2.docx]

**Multimedia Appendix 2**

Experience with the ICOnnecta’t system subjective evaluations.

***Questions***

GENERAL USAGE:

a. Overall, what has been your experience?

- - Ensure you mention wearables (watch and thermometer), and the ICOnnecta’t app.

1. POSITIVE ASPECTS:

a. What has been most beneficial to you, and how have you utilized it?

b. Do you believe using the devices has helped you better monitor your health?

c. Would you say having a record of your activity has motivated you to make any lifestyle changes?

1. AREAS TO IMPROVE:

a. Have you encountered any difficulties using the app or devices?

b. What have you missed? How could it be improved? Would you change or add anything?

1. CLOSING THOUGHTS:

a. When have the devices been most useful to you? Would you recommend them to other patients? Why?

***Answers***

User 1

- Finds motivation in observing recorded steps for increased walking.
- Views it as a personal challenge and feels competitive.
- Reflects on days with reduced walking, attributing it to factors like feeling unwell.

User 2

- Explains ease of use for ICONNECTA’T and Withings apps.
- Emphasizes the utility of the watch in visualizing variables, especially for monitoring prescribed activity.
- Expresses overall satisfaction with no specific improvement suggestions.
- Finds the post-discharge record useful and is willing to recommend it.
- Primarily uses ICOnnecta’t for questionnaires, no feedback on symptom block.

User 3

- Describes easy app usage, enhancing security and symptom control.
- Identifies no need for improvements.
- Recommends the system.
- Expresses a wish for widespread accessibility.

User 4

- Finds temperature monitoring and record-keeping highly useful.
- Views the step record as motivation to walk, framing each day as a personal challenge.
- Believes the system could be useful for others and recommends it.
- Initially faced temperature recording challenges, resolved with no further issues.
- Suggests no changes to the app.

User 5

- Commends thermometer design and temperature tracking.
- Emphasizes motivation from step counting.
- Reports no app engagement, citing a psychological lack of necessity.
- Recommends the system despite limited tech proficiency.
- Suggests a need for more technical support.

User 6

- Finds step tracking very useful, motivating increased walking.
- Despite personal inconsistency, step record compels more walking.
- Reports no device issues.
- Recommends the system for positive behavioral change.
- Primarily uses Withings app, ICOnnecta’t for questionnaires.
- Desires continued watch usage.

User 7

- Expresses high satisfaction with the watch, feeling more control over health metrics and increased motivation for walking.
- Suggests adding messaging in the app for direct communication or reporting symptoms.
- Would recommend the system to others.
- Considers purchasing the same watch.
